# Translating Exercise Benefits Over Telehealth

**DOI:** 10.1093/geroni/igaa057.2787

**Published:** 2020-12-16

**Authors:** Kalpana Padala, Shelly Lensing, Tammy Evans, Megan Pearson, Miriam Morey, Prasad Padala

**Affiliations:** 1 Central Arkansas Veterans Healthcare System, North Little Rock, Arkansas, United States; 2 University of Arkansas for Medical Sciences, Little Rock, Arkansas, United States; 3 GRECC, Central Arkansas Veterans Healthcare System, North Little Rock, Arkansas, United States; 4 Veteran Affairs Health Care System, Durham, North Carolina, United States; 5 Veterans Health Administration, Durham, North Carolina, United States; 6 Central Arkansas Veterans Healthcare System, Little Rock, Arkansas, United States

## Abstract

Background: Gerofit-Geriatric walking clinic is a home-based program that helps older veterans engage in regular walking via different platforms to improve access. The objective was to compare the outcomes of face-to-face visits to telehealth visits. Methods: Older Veterans (N=646) and walking-buddies (N=154) were seen either face-to-face or via telehealth at baseline, 2-, and 6-months. The primary intervention, pedometer feedback and motivational phone calls were delivered remotely. Results: Demographic data were similar in both Veteran groups and 47% were seen via telehealth. Compared to face-to-face, a higher proportion in the telehealth group had walking buddies (10% vs. 27%; p<0.001), received exercise counseling (75% vs. 95%; p=0.001), and reported perceived barriers (40% vs. 67%; p=0.004). There were statistically significant improvements in step-counts at 2- and 6-months compared to baseline (57% and 99% improvement; p<0.01) with no significant between-group differences. Conclusion: Tailored activity promotion programs via telehealth are effective in reaching older veterans.

